# 6-Chloro-4-(4-methyl­phen­oxy­meth­yl)-2*H*-chromen-2-one

**DOI:** 10.1107/S1600536811019258

**Published:** 2011-06-18

**Authors:** Ramakrishna Gowda, K.V. Arjuna Gowda, Mahantesha Basanagouda, Manohar V. Kulkarni

**Affiliations:** a Department of Physics, Govt. College for Women, Kolar 563 101, Karnataka, India; b Department of Physics, Govt. College for Women, Mandya 571 401, Karnataka, India; c Department of Chemistry, Karnatak University, Dharwad 580 003, Karnataka, India

## Abstract

In the title compound, C_17_H_13_ClO_3_, the coumarin and phen­oxy moieties are essentially co-planar, making a dihedral angle of 1.99 (7)°. The phen­oxy moiety is oriented anti­periplanar with respect to the coumarin ring as indicated by the C—C—O—C angle of −179.97 (16)°. In the crystal, the sheet-like packing is stabilized by inter­molecular C—H⋯O and C—H⋯Cl hydrogen bonds.

## Related literature

For the structure of 7-methyl-4-tolyl­oxymethyl­coumarin, see: Vasudevan *et al.* (1990 ▶).
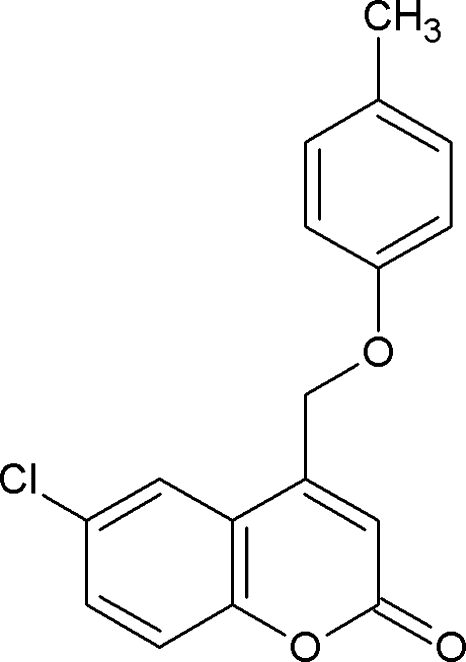


## Experimental

### 

#### Crystal data

C_17_H_13_ClO_3_
*M* *_r_* = 300.72
Monoclinic, 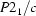

*a* = 15.3068 (5) Å
*b* = 6.9353 (2) Å
*c* = 14.9566 (5) Å
β = 116.923 (2)°
*V* = 1415.66 (8) Å^3^
*Z* = 4
Mo *K*α radiation
μ = 0.28 mm^−1^
*T* = 293 K
0.30 × 0.20 × 0.20 mm

#### Data collection

Bruker Kappa APEXII CCD diffractometer
Absorption correction: multi-scan (*SADABS*; Bruker, 2004 ▶) *T*_min_ = 0.932, *T*_max_ = 0.967
16899 measured reflections
3818 independent reflections
2448 reflections with *I* > 2σ(*I*)
*R*_int_ = 0.038

#### Refinement

*R*[*F* ^2^ > 2σ(*F* ^2^)] = 0.053
*wR*(*F* ^2^) = 0.170
*S* = 1.08
3818 reflections
191 parameters
H-atom parameters constrained
Δρ_max_ = 0.27 e Å^−3^
Δρ_min_ = −0.22 e Å^−3^

### 

Data collection: *APEX2* (Bruker, 2004 ▶); cell refinement: *APEX2* and *SAINT* (Bruker, 2004 ▶); data reduction: *SAINT* and *XPREP* (Bruker, 2004 ▶); program(s) used to solve structure: *SIR92* (Altomare *et al.*, 1994 ▶); program(s) used to refine structure: *SHELXL97* (Sheldrick, 2008 ▶); molecular graphics: *ORTEP-3* (Farrugia, 1997 ▶) and *Mercury* (Macrae *et al.*, 2006 ▶); software used to prepare material for publication: *SHELXL97*.

## Supplementary Material

Crystal structure: contains datablock(s) global, I. DOI: 10.1107/S1600536811019258/vm2090sup1.cif

Structure factors: contains datablock(s) I. DOI: 10.1107/S1600536811019258/vm2090Isup2.hkl

Supplementary material file. DOI: 10.1107/S1600536811019258/vm2090Isup3.cml

Additional supplementary materials:  crystallographic information; 3D view; checkCIF report

## Figures and Tables

**Table 1:** Hydrogen-bond geometry (Å, °)

*D*—H⋯*A*	*D*—H	H⋯*A*	*D*⋯*A*	*D*—H⋯*A*
C10—H10*B*⋯O2^i^	0.97	2.47	3.303 (5)	143
C4—H4⋯O2^i^	0.93	2.69	3.553 (4)	154
C1—H1⋯Cl1^ii^	0.93	2.88	3.693 (4)	146

Symmetry codes: (i) 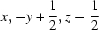; (ii) 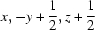.

**Fig. 1.**
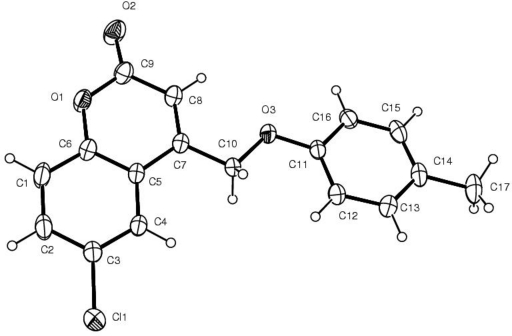
The molecular structure of the title compound, showing 50% probability displacement ellipsoids and the atomic numbering.

**Fig. 2.**
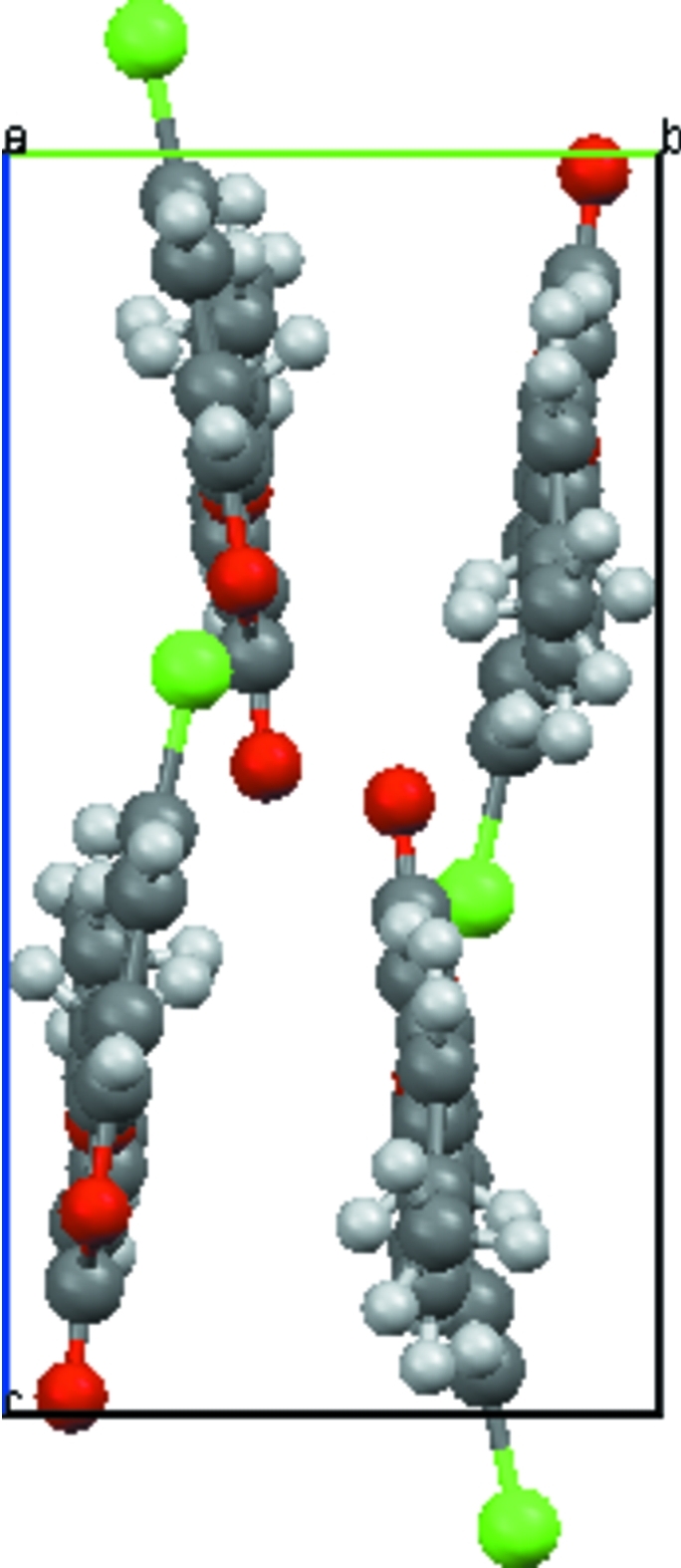
Packing diagram viewed down a axis and molecules oriented as parallel layers along c axis.

**Fig. 3.**
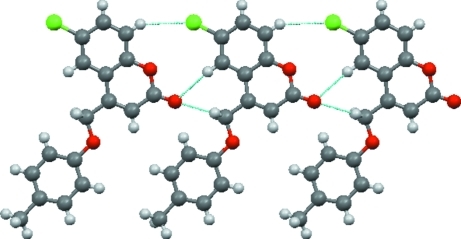
Packing diagram showing C—H···O and C—H···Cl hydrogen bonding.

## supplementary crystallographic information

### Comment

The first report on X-ray diffraction studies on 4-aryloxymethylcoumarins has revealed that in solid state the molecules exist as head-tail dimers as observed in the case of 7-methyl-4-tolyloxymethylcoumarin (Vasudevan *et al.*, 1990). In the light of these observations a chloro substituted 4-aryloxymethylcoumarin has been subjected to X-ray diffraction studies. A significant bond deviation is observed at C5—C7 (1.449 (2) Å) due to the bridging of α-pyrone and benzene ring at C5 and the substituent present at C7. This is also reflected at C8—C9 and C7—C10 due to the presence of O2 at C9 and a phenoxy group at C10, respectively. Significant bond angle deviations are observed at C6—C5—C4 (117.91 (17)°) and C6—C5—C7 (117.61 (18)°). Another significant bond angle deviation is observed at C15—C14—C13 (117.46 (18)°) due to presence of the electron donating methyl group on C14. The molecules are oriented as parallel layers along the *c* axis as shown in Fig 2. The sheet-like packing is stabilized by intermolecular C—H···O and C—H···Cl hydrogen bonds (Table 1, Fig. 3).

### Experimental

A mixture of 4-methyl-phenol (10 mmol) and anhydrous potassium carbonate (10 mmol) was stirred for 30 minutes in dry acetone (30 ml). To this, 6-chloro-4-bromomethylcoumarin (10 mmol) was added and the stirring was continued for 24 h. Then, the resulting reaction mixture was poured to crushed ice. The separated solid was filtered and washed with 1:1 HCl (30 ml) and with water. Then product 6-chloro-4-[(4-methyl)phenoxymethyl]coumarin was recrystallized from ethyl acetate.

### Refinement

Hydrogen atoms were positioned geometrically with C—H = 0.93–0.97 A° and included in the refinment in a riding-model approximation with *U**_iso_*(H) = 1.2 *U**_eq_*(C) or 1.5 *U**_eq_*(C) for methyl C atoms.

### Crystal data

**Table:**

C_17_H_13_ClO_3_	*F*(000) = 624
*M**_r_* = 300.72	*D*_x_ = 1.411 Mg m^−^^3^
Monoclinic, *P*2_1_/*c*	Mo *K*α radiation, λ = 0.71073 Å
Hall symbol: -P 2ybc	Cell parameters from 4071 reflections
*a* = 15.3068 (5) Å	θ = 2.7–28.3°
*b* = 6.9353 (2) Å	µ = 0.28 mm^−^^1^
*c* = 14.9566 (5) Å	*T* = 293 K
β = 116.923 (2)°	Block, colourless
*V* = 1415.66 (8) Å^3^	0.30 × 0.20 × 0.20 mm
*Z* = 4	

### Data collection

**Table:**

Bruker Kappa APEXII CCD diffractometer	3818 independent reflections
Radiation source: fine-focus sealed tube	2448 reflections with *I* > 2σ(*I*)
graphite	*R*_int_ = 0.038
ω and φ scans	θ_max_ = 29.2°, θ_min_ = 1.5°
Absorption correction: multi-scan (*SADABS*; Bruker, 2004)	*h* = −20→20
*T*_min_ = 0.932, *T*_max_ = 0.967	*k* = −9→6
16899 measured reflections	*l* = −20→20

### Refinement

**Table:**

Refinement on *F*^2^	Primary atom site location: structure-invariant direct methods
Least-squares matrix: full	Secondary atom site location: difference Fourier map
*R*[*F*^2^ > 2σ(*F*^2^)] = 0.053	Hydrogen site location: inferred from neighbouring sites
*wR*(*F*^2^) = 0.170	H-atom parameters constrained
*S* = 1.08	*w* = 1/[σ^2^(*F*_o_^2^) + (0.0833*P*)^2^ + 0.2177*P*] where *P* = (*F*_o_^2^ + 2*F*_c_^2^)/3
3818 reflections	(Δ/σ)_max_ < 0.001
191 parameters	Δρ_max_ = 0.27 e Å^−^^3^
0 restraints	Δρ_min_ = −0.22 e Å^−^^3^

### Special details

**Table:**

Geometry. All esds (except the esd in the dihedral angle between two l.s. planes) are estimated using the full covariance matrix. The cell esds are taken into account individually in the estimation of esds in distances, angles and torsion angles; correlations between esds in cell parameters are only used when they are defined by crystal symmetry. An approximate (isotropic) treatment of cell esds is used for estimating esds involving l.s. planes.
Refinement. Refinement of F^2^ against ALL reflections. The weighted R-factor wR and goodness of fit S are based on F^2^, conventional R-factors R are based on F, with F set to zero for negative F^2^. The threshold expression of F^2^ > 2sigma(F^2^) is used only for calculating R-factors(gt) etc. and is not relevant to the choice of reflections for refinement. R-factors based on F^2^ are statistically about twice as large as those based on F, and R- factors based on ALL data will be even larger.

### Fractional atomic coordinates and isotropic or equivalent isotropic displacement parameters (Å^2^)

**Table:**

	*x*	*y*	*z*	*U*_iso_*/*U*_eq_	
C1	0.09025 (16)	0.1827 (4)	0.68708 (19)	0.0627 (7)	
H1	0.0528	0.1718	0.7215	0.075*	
C2	0.04581 (17)	0.2170 (4)	0.5863 (2)	0.0652 (7)	
H2	−0.0219	0.2299	0.5514	0.078*	
C3	0.10297 (15)	0.2323 (4)	0.53657 (16)	0.0509 (5)	
C4	0.20280 (14)	0.2106 (3)	0.58555 (15)	0.0430 (5)	
H4	0.2395	0.2191	0.5503	0.052*	
C5	0.24892 (13)	0.1756 (3)	0.68853 (14)	0.0371 (4)	
C6	0.19059 (15)	0.1644 (3)	0.73768 (16)	0.0448 (5)	
C7	0.35374 (13)	0.1530 (3)	0.74835 (14)	0.0359 (4)	
C8	0.39041 (14)	0.1275 (3)	0.84735 (15)	0.0428 (5)	
H8	0.4578	0.1139	0.8853	0.051*	
C9	0.32906 (15)	0.1204 (3)	0.89761 (16)	0.0482 (5)	
C10	0.41673 (12)	0.1566 (3)	0.69576 (14)	0.0384 (4)	
H10A	0.4010	0.0477	0.6503	0.046*	
H10B	0.4049	0.2741	0.6568	0.046*	
C11	0.58438 (13)	0.1494 (3)	0.73188 (15)	0.0380 (4)	
C12	0.56245 (14)	0.1461 (3)	0.63200 (15)	0.0437 (5)	
H12	0.4976	0.1427	0.5829	0.052*	
C13	0.63822 (15)	0.1481 (3)	0.60532 (17)	0.0488 (5)	
H13	0.6233	0.1448	0.5377	0.059*	
C14	0.73516 (15)	0.1549 (3)	0.67619 (18)	0.0505 (5)	
C15	0.75450 (15)	0.1587 (3)	0.77520 (19)	0.0525 (6)	
H15	0.8193	0.1637	0.8243	0.063*	
C16	0.68085 (14)	0.1553 (3)	0.80433 (16)	0.0458 (5)	
H16	0.6960	0.1571	0.8720	0.055*	
C17	0.81634 (18)	0.1592 (4)	0.6449 (2)	0.0747 (8)	
H17A	0.8295	0.2904	0.6344	0.112*	
H17B	0.7965	0.0876	0.5839	0.112*	
H17C	0.8745	0.1026	0.6967	0.112*	
O1	0.23009 (10)	0.1370 (2)	0.83898 (11)	0.0524 (4)	
O2	0.35626 (12)	0.1007 (3)	0.98589 (11)	0.0684 (5)	
O3	0.51603 (9)	0.1474 (2)	0.76774 (10)	0.0459 (4)	
Cl1	0.04586 (4)	0.28400 (13)	0.40961 (5)	0.0790 (3)	

### Atomic displacement parameters (Å^2^)

**Table:**

	*U*^11^	*U*^22^	*U*^33^	*U*^12^	*U*^13^	*U*^23^
C1	0.0443 (12)	0.093 (2)	0.0644 (14)	−0.0023 (12)	0.0368 (11)	−0.0043 (13)
C2	0.0363 (10)	0.099 (2)	0.0655 (15)	0.0039 (12)	0.0278 (10)	−0.0017 (14)
C3	0.0389 (10)	0.0683 (15)	0.0476 (11)	0.0036 (10)	0.0213 (9)	0.0021 (10)
C4	0.0393 (10)	0.0470 (12)	0.0499 (11)	0.0003 (9)	0.0266 (9)	−0.0001 (9)
C5	0.0359 (9)	0.0345 (10)	0.0472 (10)	−0.0020 (7)	0.0243 (8)	−0.0036 (8)
C6	0.0444 (10)	0.0494 (13)	0.0495 (11)	−0.0037 (9)	0.0291 (9)	−0.0048 (9)
C7	0.0382 (9)	0.0289 (9)	0.0473 (10)	−0.0025 (7)	0.0252 (8)	−0.0040 (8)
C8	0.0407 (10)	0.0450 (12)	0.0470 (11)	−0.0061 (8)	0.0236 (8)	−0.0072 (9)
C9	0.0512 (11)	0.0538 (14)	0.0463 (11)	−0.0096 (10)	0.0278 (9)	−0.0128 (10)
C10	0.0314 (8)	0.0445 (11)	0.0413 (9)	0.0016 (8)	0.0183 (7)	0.0024 (8)
C11	0.0337 (9)	0.0344 (10)	0.0497 (10)	0.0020 (7)	0.0223 (8)	0.0023 (8)
C12	0.0342 (9)	0.0480 (12)	0.0501 (11)	−0.0015 (8)	0.0201 (8)	−0.0021 (9)
C13	0.0473 (11)	0.0518 (13)	0.0556 (12)	−0.0010 (10)	0.0308 (10)	−0.0020 (10)
C14	0.0416 (11)	0.0450 (12)	0.0747 (15)	0.0031 (9)	0.0351 (11)	−0.0021 (11)
C15	0.0299 (9)	0.0511 (13)	0.0724 (15)	0.0033 (9)	0.0195 (9)	0.0012 (11)
C16	0.0387 (10)	0.0475 (12)	0.0492 (11)	0.0051 (9)	0.0181 (8)	0.0031 (9)
C17	0.0522 (14)	0.084 (2)	0.108 (2)	0.0047 (13)	0.0538 (15)	−0.0040 (16)
O1	0.0474 (8)	0.0715 (11)	0.0489 (8)	−0.0054 (7)	0.0310 (7)	−0.0060 (7)
O2	0.0664 (10)	0.1026 (15)	0.0437 (9)	−0.0135 (10)	0.0315 (8)	−0.0150 (9)
O3	0.0328 (7)	0.0636 (10)	0.0443 (8)	0.0027 (6)	0.0201 (6)	0.0057 (6)
Cl1	0.0481 (3)	0.1308 (7)	0.0550 (4)	0.0162 (3)	0.0206 (3)	0.0183 (4)

### Geometric parameters (Å, °)

**Table:**

C1—C2	1.365 (3)	C10—O3	1.411 (2)
C1—C6	1.377 (3)	C10—H10A	0.9700
C1—H1	0.9300	C10—H10B	0.9700
C2—C3	1.385 (3)	C11—O3	1.375 (2)
C2—H2	0.9300	C11—C12	1.375 (3)
C3—C4	1.371 (3)	C11—C16	1.381 (3)
C3—Cl1	1.731 (2)	C12—C13	1.387 (3)
C4—C5	1.394 (3)	C12—H12	0.9300
C4—H4	0.9300	C13—C14	1.380 (3)
C5—C6	1.392 (2)	C13—H13	0.9300
C5—C7	1.449 (2)	C14—C15	1.373 (3)
C6—O1	1.367 (2)	C14—C17	1.513 (3)
C7—C8	1.336 (3)	C15—C16	1.381 (3)
C7—C10	1.495 (2)	C15—H15	0.9300
C8—C9	1.445 (3)	C16—H16	0.9300
C8—H8	0.9300	C17—H17A	0.9600
C9—O2	1.199 (2)	C17—H17B	0.9600
C9—O1	1.369 (3)	C17—H17C	0.9600
			
C2—C1—C6	119.81 (19)	O3—C10—H10B	109.9
C2—C1—H1	120.1	C7—C10—H10B	109.9
C6—C1—H1	120.1	H10A—C10—H10B	108.3
C1—C2—C3	119.1 (2)	O3—C11—C12	124.69 (16)
C1—C2—H2	120.5	O3—C11—C16	115.23 (17)
C3—C2—H2	120.5	C12—C11—C16	120.08 (17)
C4—C3—C2	121.8 (2)	C11—C12—C13	119.20 (18)
C4—C3—Cl1	119.73 (16)	C11—C12—H12	120.4
C2—C3—Cl1	118.47 (17)	C13—C12—H12	120.4
C3—C4—C5	119.56 (18)	C14—C13—C12	121.9 (2)
C3—C4—H4	120.2	C14—C13—H13	119.1
C5—C4—H4	120.2	C12—C13—H13	119.1
C6—C5—C4	117.91 (17)	C15—C14—C13	117.46 (18)
C6—C5—C7	117.61 (18)	C15—C14—C17	121.8 (2)
C4—C5—C7	124.48 (16)	C13—C14—C17	120.7 (2)
O1—C6—C1	116.47 (17)	C14—C15—C16	122.1 (2)
O1—C6—C5	121.71 (18)	C14—C15—H15	118.9
C1—C6—C5	121.8 (2)	C16—C15—H15	118.9
C8—C7—C5	119.38 (16)	C15—C16—C11	119.2 (2)
C8—C7—C10	122.52 (17)	C15—C16—H16	120.4
C5—C7—C10	118.09 (16)	C11—C16—H16	120.4
C7—C8—C9	122.34 (18)	C14—C17—H17A	109.5
C7—C8—H8	118.8	C14—C17—H17B	109.5
C9—C8—H8	118.8	H17A—C17—H17B	109.5
O2—C9—O1	116.54 (18)	C14—C17—H17C	109.5
O2—C9—C8	126.4 (2)	H17A—C17—H17C	109.5
O1—C9—C8	117.07 (17)	H17B—C17—H17C	109.5
O3—C10—C7	109.05 (15)	C6—O1—C9	121.85 (15)
O3—C10—H10A	109.9	C11—O3—C10	116.67 (14)
C7—C10—H10A	109.9		
			
C6—C1—C2—C3	0.1 (4)	C7—C8—C9—O1	1.3 (3)
C1—C2—C3—C4	1.2 (4)	C8—C7—C10—O3	−5.3 (3)
C1—C2—C3—Cl1	−177.8 (2)	C5—C7—C10—O3	175.47 (16)
C2—C3—C4—C5	−1.2 (4)	O3—C11—C12—C13	−179.84 (19)
Cl1—C3—C4—C5	177.74 (16)	C16—C11—C12—C13	0.3 (3)
C3—C4—C5—C6	0.0 (3)	C11—C12—C13—C14	−0.5 (3)
C3—C4—C5—C7	−179.1 (2)	C12—C13—C14—C15	0.3 (3)
C2—C1—C6—O1	177.8 (2)	C12—C13—C14—C17	−179.3 (2)
C2—C1—C6—C5	−1.4 (4)	C13—C14—C15—C16	0.2 (3)
C4—C5—C6—O1	−177.77 (18)	C17—C14—C15—C16	179.8 (2)
C7—C5—C6—O1	1.4 (3)	C14—C15—C16—C11	−0.5 (3)
C4—C5—C6—C1	1.3 (3)	O3—C11—C16—C15	−179.67 (19)
C7—C5—C6—C1	−179.5 (2)	C12—C11—C16—C15	0.2 (3)
C6—C5—C7—C8	−1.7 (3)	C1—C6—O1—C9	−178.8 (2)
C4—C5—C7—C8	177.35 (19)	C5—C6—O1—C9	0.4 (3)
C6—C5—C7—C10	177.49 (17)	O2—C9—O1—C6	178.5 (2)
C4—C5—C7—C10	−3.4 (3)	C8—C9—O1—C6	−1.7 (3)
C5—C7—C8—C9	0.4 (3)	C12—C11—O3—C10	−4.7 (3)
C10—C7—C8—C9	−178.76 (19)	C16—C11—O3—C10	175.20 (17)
C7—C8—C9—O2	−178.9 (2)	C7—C10—O3—C11	−179.97 (16)

### Hydrogen-bond geometry (Å, °)

**Table:**

*D*—H···*A*	*D*—H	H···*A*	*D*···*A*	*D*—H···*A*
C10—H10B···O2^i^	0.97	2.47	3.303 (5)	143
C4—H4···O2^i^	0.93	2.69	3.553 (4)	154
C1—H1···Cl1^ii^	0.93	2.88	3.693 (4)	146

Symmetry codes: (i) *x*, −*y*+1/2, *z*−1/2; (ii) *x*, −*y*+1/2, *z*+1/2.
